# Text Messaging Intervention for Mental Wellness in American Indian and Alaska Native Teens and Young Adults (BRAVE Study): Analysis of User Engagement Patterns

**DOI:** 10.2196/32138

**Published:** 2022-02-25

**Authors:** Julia Wrobel, Joshva Silvasstar, Roger Peterson, Kanku Sumbundu, Allyson Kelley, David Stephens, Stephanie Craig Rushing, Sheana Bull

**Affiliations:** 1 Colorado School of Public Health University of Colorado Aurora, CO United States; 2 Northwest Portland Area Indian Health Board Portland, OR United States; 3 Allyson Kelley and Associates PLLC Sisters, OR United States

**Keywords:** American Indian, Alaska Native, adolescent, mental health, help-seeking skills, text messaging, mHealth, behavioral intervention, user engagement, feasibility, engagement, low-touch, intervention

## Abstract

**Background:**

Many American Indian and Alaska Native (AI/AN or Native) communities express concern about high rates of suicide and poor mental health. Technology-based health interventions that nurture resilience, coping skills, connectedness, and help-seeking skills may be an effective strategy for promoting health and wellbeing among AI/AN youth. The Northwest Portland Area Indian Health Board designed the BRAVE intervention for AI/AN youth. BRAVE is delivered via SMS text messaging and includes role model videos, mental wellness strategies, links to culturally relevant resources, and social support from family and friends.

**Objective:**

The aim of this study is to explore system data from the BRAVE intervention to determine patterns of user engagement and differences in psychosocial outcomes based on the number of clicks on BRAVE content.

**Methods:**

The BRAVE study included 1030 AI/AN teens and young adults nationwide (15 to 24 years old). The message series in the BRAVE and STEM study arms included 3 to 5 SMS text messages per week, featuring 1 role model video and 1 image per week. Messages were sent out via Mobile Commons (Upland Software Inc), a mobile messaging provider that supports text, picture, and video SMS.

**Results:**

Of the 509 participants in the original BRAVE analysis, 270 had sufficient data to analyze user engagement, with at least 1 trackable click on a study SMS text message. Of the 270, 184 (68.1%) were female, 50 (18.5%) were male, and 36 (13.3%) selected another gender category. The average participant was 20.6 years old, with a minimum and maximum age of 15 and 26 years. Most participants had relatively low engagement measured by the number of clicks (median 2; mean 3.4), although others clicked message content as many as 49 times. Users engaged most frequently with the YouTube-based content (viewing 1 of 7 role model videos), with 64.8% (175/270) of total clicks coming from the role model videos, and earlier episodes receiving the highest number of clicks. Most baseline psychosocial measures were not significantly associated with the number of links clicked. However, help-seeking behavior was highly significant (*P*<.001), with a rate ratio of 0.82 (0.73, 0.92), indicating that each 1-unit increase in help-seeking score at baseline was associated with an 18% decrease in the expected number of study content clicks.

**Conclusions:**

This is the first study to set initial standards for assessing user engagement in an mHealth intervention. Our work underscores the feasibility of exploring the impact of engagement on intended outcomes, allowing for more precise exploration of the dose-response relationship that may be realized through these low-touch interventions that offer promising potential for reaching high numbers of program participants.

**Trial Registration:**

ClinicalTrials.gov NCT04979481; https://clinicaltrials.gov/ct2/show/NCT04979481

## Introduction

### Background

In the United States, many youths face a mental health crisis, with 1 of every 5 considering suicide each year and 1 million attempting suicide [1], now the third leading cause of death for this age group [2]. The teen years are frequently cited as the time when suicidal ideation begins, which underscores an urgent need to support mental health for teens [3,4]. Suicide prevention and mental health promotion remain a critical challenge for American Indian and Alaska Native (AI/AN or Native) communities, in particular [5]. Among AI/AN youth in the 9th to 12th grade, the past-year prevalence of suicidal thoughts, planning, and attempts was nearly 15% in 2017 [6]. Suicide was the second leading cause of death for Native youth aged 10 to 24 years, a rate that is 2.5 times higher than the national average [7].

Multilevel interventions are critically needed to build protective factors against suicide and violence [8]. According to the recent report, Culture Forward, key factors that protect against suicide include hope, self-efficacy, connectedness to family, community belonging, identity and participation in tribal culture, family living a traditional lifestyle, self-determination, spirituality, connectedness to community and lands, and talking to family and friends about problems [9]. Technology-based health interventions that nurture self-esteem, help-seeking skills, and connectedness to their self, peers, family, community, and the natural environment may be an effective strategy for promoting health and wellbeing for AI/AN youth.

### Mobile and Digital Technologies

There is growing evidence of the efficacy of interventions that use mobile and digital technologies to support healthy behaviors, including mental health [10], although efficacy varies across text message programs. Researchers in this domain have limited information and agreement on optimal strategies for designing engaging SMS text message content, measuring engagement, if the timing and dose of messages moderates or mediates outcomes, or if there is a specific threshold for engagement to realize intervention effects.

We know from prior research in mobile and digital health that attention to the design and content of text messages [11] is important to increase engagement with messaging, and that health communication theory can be employed to frame text messages in a way that increases their resonance for diverse demographics (eg, various genders, races, ethnicities) [12]. A meta-analysis of health promotion interventions relying on text messages supported this notion, demonstrating that targeted messages (ie, generating messages that resonate for a specific demographic such as men, younger adults, African American individuals, or Latino individuals) combined with tailored messages (ie, making content specific to individuals based on information they provide), produce significantly greater effects compared to more generic messages [13].

Researchers have used various approaches to measure engagement with mobile and digital health interventions, including in-depth interviews with users, ecological momentary assessment (EMA), and reviews of system use data [14]. However, currently, there is a lack of agreement on the best strategy to measure participants’ engagement with health-related text messages. EMA, which deploys brief surveys to occasionally poll users about engagement, requires users to complete the surveys to document their engagement, so although useful, this may be challenging to implement. Another strategy is to rely on backend user data to document engagement, a more passive and potentially less cumbersome approach.

The consideration of dose and the appropriate level of engagement with SMS text messaging is important given assumptions that a certain threshold of engagement is necessary to generate intervention effects [15-17], and evidence that participants demonstrate waning interest in text messages over time, sometimes precipitously [18-22].

In this paper, we focus on a review of system data for a study conducted to assess the efficacy of an SMS text messaging intervention to promote mental wellness in AI/AN teens and young adults. Prior papers have focused on the formative design of the intervention, recruitment methods, and the efficacy of the BRAVE intervention [23,24]. Here, we analyze an array of patterns in user engagement using passively collected backend user data. Our objectives are to understand which content was most popular, analyze the timing of messaging and engagement, and test the association between high engagement and efficacy outcomes.

## Methods

### Research Partners

The BRAVE intervention was designed by the THRIVE and We R Native adolescent health teams at the Northwest Portland Area Indian Health Board (NPAIHB). The NPAIHB is a regional, tribal nonprofit organization that represents 43 federally recognized tribes in Washington, Oregon, and Idaho. The Northwest Tribal Epidemiology Center is housed under NPAIHB and provides support through research, surveillance, and public health capacity building in partnership with the Northwest Tribes. NPAIHB partnered with the mHealth Impact Lab at the Colorado School of Public Health to develop, implement, and evaluate the BRAVE intervention. NPAIHB recruited study participants and delivered SMS text messages, and the mHealth Impact Lab led the design of data collection tools, data collection, and analysis. The partnership was supported by the Technology & Adolescent Mental Wellness (TAM) program, run by the Social Media and Adolescent Health Research Team and housed within the Department of Pediatrics at the University of Wisconsin-Madison.

### Study Overview, Population, and Recruitment

All data collection methods were approved by the Portland Area Indian Health Service Institutional Review Board in Portland, Oregon (1384639). All instruments and data collection methods were reviewed and approved by the institutional review board before data collection took place.

Youth who enrolled in the study received either 8 weeks of BRAVE text messages or 8 weeks of STEM text messages, then crossed over to the other arm and received the next set of messages. The message series in both study arms included 3 to 5 text messages per week, featuring 1 role model video and 1 image per week.

The BRAVE intervention was designed to amplify and reinforce healthy social norms and cultural values, teach suicide warning signs, prepare youth to initiate difficult conversations with peers and trusted adults, encourage youth to access mental health resources (ie, tribal clinics, chat lines), destigmatize mental health services, and connect youth to trusted adults. The message series included links to 7 role model videos (1 to 3 minutes long each) that featured relatable characters experiencing and addressing violent behavior, alcohol misuse, and suicidality (through the eyes of a perpetrator, an intimate partner violence survivor, and a peer bystander), intended to demonstrate important coping strategies and help-seeking skills.

### Participant Recruitment and Eligibility

Participant recruitment occurred via We R Native’s social media channels (Facebook and Instagram, including text messaging on these platforms) and listservs associated with tribes, tribal health organizations, Indian education, and human service organizations that serve AI/AN teens and young adults. Youth were asked to text the keyword BRAVE to a short code, which triggered a series of eligibility and consent text messages, including a link to a web-based consent form for more information about this study.

**Figure 1:**
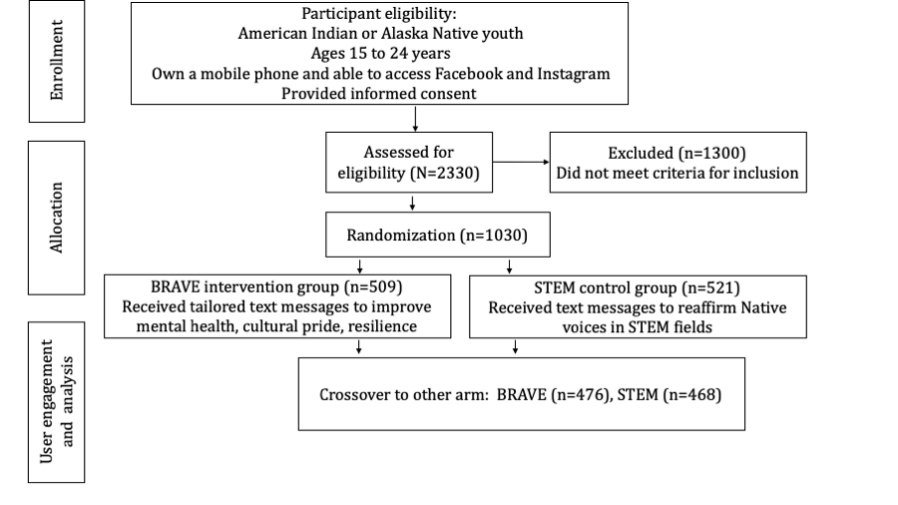
Flowchart summarizing participant recruitment and randomization.

The study included self-identified AI/AN youth ages 15 to 24 years. All participants were required to have a cell phone with text message capabilities. To enroll, participants were required to complete the presurvey. Those who met the eligibility requirements and completed the presurvey were randomly assigned to a study arm (n=1030) and invited to complete a baseline survey and 3 follow-up surveys. Participants received a US $10 amazon gift code for each survey they completed, for a compensation of up to US $40 per person in appreciation for their time. Figure 1 summarizes participant recruitment, randomization, and user engagement.

### Delivery of BRAVE Messages

For consistency across the 2 study arms, the regular intervention text messages were scheduled to go out in the evenings (between 4 PM and 6 PM EDT). The messages were sent out via Mobile Commons (Upland Software Inc), a mobile messaging provider that supports text, picture, and video SMS [25]. Mobile Commons allows users to track both message delivery and message engagement (ie, link clicks).

To analyze message delivery, we tracked when each message was delivered, its content, and the participant’s phone number. Mobile Commons also tracks message engagement by generating a record each time a study participant clicks a link from an SMS text message. For each record we stored the exact time the message was clicked, the message content, and the participant’s phone number and IP address.

### Data Sets

The Mobile Commons engagement data required substantial data cleaning, which is described below. We produced 2 data sets for analysis. The first data set examined the relationship between the timing of message delivery and timing of message or link clicks for all messages, where link click and message click are used interchangeably and define an event where a subject clicked the link within a message. The second data set merges the engagement data with demographic (gender, age) and efficacy data from the BRAVE study. This enabled us to analyze the number of clicks by gender, message content, and psychosocial health outcomes. All participants were required to complete the baseline survey at the time of enrollment. Enrollees were asked to complete the same survey after the first set of messages. At the end of the intervention period (2 months), participants crossed over to receive the second set of messages and were asked to complete the survey a third time. The study team discontinued communication and asked participants to complete the final survey 90 days later.

### Psychosocial Survey Measures

The psychosocial survey measures were influenced by the Healing of the Canoe Survey as part of the Tribal Health: Reaching out InVolves Everyone project [26] and taken from validated survey tools, including the Youth Risk Behavior Surveillance Survey [27], the Youth Coping Responses Inventory [28], the Child and Youth Resilience Measure [29], the Bandura Self-Efficacy Beliefs of Adolescents Scale [30], the Counseling and Help Seeking Questionnaire [31], and the Rosenberg Self-Esteem Scale [32]. Outcomes of interest included health, help-seeking behavior, identification with cultural heritage (cultural identity), self-efficacy, self-esteem, negative coping behavior (alcohol and drug misuse), positive coping behavior, and resilience. Each measure is an aggregate score calculated from multiple survey questions, as defined in [23,24].

### Analysis

All statistical analyses were completed using or R, version 4.0 (R Foundation for Statistical Computing) [33]. All statistical tests were considered significant when *P*<0.05. Descriptive statistics were calculated to summarize engagement levels, assess which message content was most popular, and compare engagement across genders. To increase the power of our analyses, we collapsed gender into three categories (male, female, and other).

The analysis of the relationship between user engagement and efficacy measures at baseline including health, resilience, coping skills, self-efficacy, self-esteem, cultural pride and identity, and help-seeking behavior was performed using Poisson regression. A separate Poisson regression model was used for each efficacy measure, with user engagement measured by the number of clicks and composite score of the efficacy measure as the model covariate of interest. We report the exponentiated coefficients and 95% confidence intervals from the Poisson regression models, which are interpreted as rate ratios.

## Results

### Participant Characteristics and Engagement

Of the 509 participants randomized to the BRAVE arm of the study after enrollment, 270 had 1 trackable click on a study-related message that could be matched to baseline BRAVE survey data using their cell phone number. The gender breakdown of the 270 participants is as follows: 184 were female (68.1%), 50 were male (18.5%), and 36 selected another gender category (13.3%). The average participant was 20.6 years old, with a minimum and maximum age of 15 and 26 years, respectively. Table 1 shows summary statistics for the number of links clicked in the data set, broken down by gender. The data are highly skewed, where most of the 270 users had relatively low engagement as measured by the number of clicks (median 2; mean 3.4), although some users clicked message links as many as 49 times.

**Table 1:** Maximum, mean, and median number of links clicked for users with at least 1 trackable link on a study-related message.

Gender category	Maximum number of clicks	Mean number of clicks	Median number of clicks
Female (n=184)	36	3.2	2
Male (n=50)	27	3.3	2
Other (n=36)	49	4.4	2
All users (n=270)	49	3.4	2

Users engaged most frequently with the YouTube-based content featuring role model videos, with 64.7% (565/873) of the total clicks derived from the BRAVE video episodes, and earlier episodes receiving the highest number of views. Of the 270 users with at least 1 trackable link, 128 (47.4%) opened the first YouTube video (Episode 1: Alex), 112 (41.5%) opened the second YouTube video (Episode 2: Chris), and 96 (35.6%) clicked a link on the We R Native webpage article about resilience. A breakdown of engagement by type of message is provided in Table 2.

**Table 2:** Count and frequency of clicks and number of unique users who accessed each type of message.^a^

Message content	Total clicks (n=873), n (%)	Number of unique users (n=270), n (%)	Mean clicks per user (maximum)
We R Native YouTube video: Episode 1: Alex	165 (18.9)	128 (47.4)	1.31 (7)
We R Native YouTube video: Episode 2: Chris	144 (16.5)	112 (41.5)	1.33 (6)
We R Native article: How Does a Person Become Resilient?	128 (14.7)	96 (35.6)	1.36 (5)
We R Native YouTube video: Episode 4: Alex	76 (8.7)	49 (18.1)	1.58 (11)
We R Native YouTube video: Episode 3: Benny	71 (8.1)	59 (21.9)	1.22 (4)
We R Native YouTube video: Episode 5: Chris	66 (7.6)	41 (15.2)	1.67 (9)
Resource article on domestic or dating violence/abuse	62 (7.1)	45 (16.7)	1.43 (8)
We R Native YouTube video: Episode 6: Benny	44 (5)	36 (13.3)	1.3 (4)
We R Native article: Creating Safe Spaces	41 (4.7)	28 (10.4)	1.66 (7)
Resource on healthy relationships and dating	34 (3.9)	27 (10)	1.29 (4)
StrongHearts Native Helpline	24 (2.7)	15 (5.6)	1.69 (6)
Tradition Not Addiction community Facebook page	19 (2.2)	16 (5.9)	1.47 (6)

^a^Numbers are provided for the total (unique users).

### User Engagement Times

**Figure 2:**
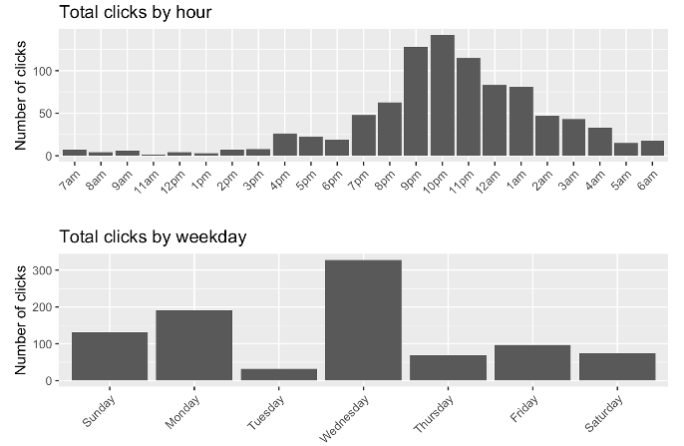
Total clicks by hour and weekday.

A time-dependent analysis of the engagement data shows that participants tend to interact with content at specific times of the day and days of the week, and are most likely to click on a message soon after it was sent. Figure 2 shows a histogram of the distribution of clicks by hour of the day (top panel) and by day of the week (bottom panel). Messages were most often clicked in the evening hours. Wednesday is the day of the week when links were most often clicked. An analysis of when users tended to interact with the content compared to when the content is sent is provided Table 3. Table 3 summarizes (in percentiles) the time elapsed (in minutes) from when a message was sent out by the BRAVE team to when a user clicked on it. For example, 50% of messages were clicked on less than 1 minute after they were sent, and 75% of messages were clicked on less than 24 hours after they were sent.

**Table 3:** Percentile of messages clicked by the time elapsed.^a^

Percentile of messages	Time elapsed
50	<1 minute
75	<24 hours
90	<100 hours
99	<37 days

^a^Time elapsed was calculated based on when the message was sent and when the message was clicked.

**Figure 3:**
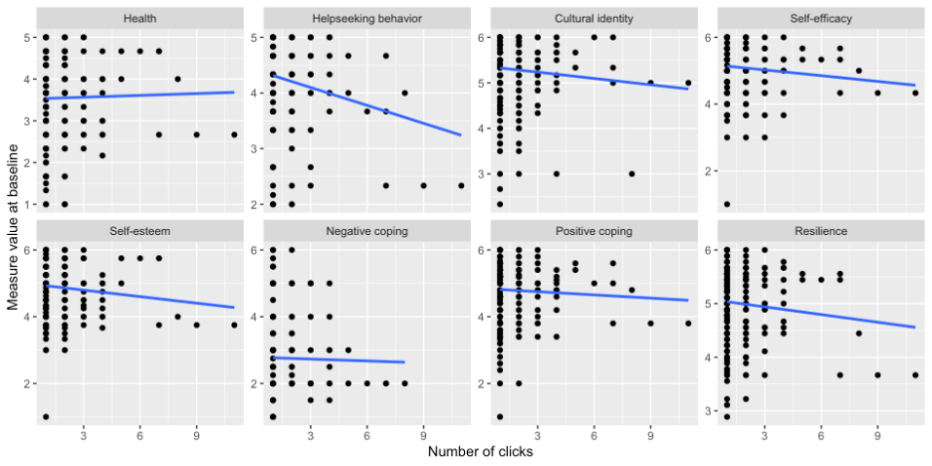
Scatter plots of the composite score at baseline for each efficacy measure against the number of clicks.

Finally, we analyzed the correlation between the number of links clicked and composite scores for the psychosocial outcome measures described in the BRAVE efficacy paper [23,24]. The measure scores are on a continuous scale of 1 to 5, which is a composite score across survey questions that were used and reported in BRAVE efficacy paper [23,24]. Plots of composite scores at baseline for each efficacy measure against the number of clicks are shown in Figure 2. The blue line indicates a linear least squares fit through the data. In these plots, although the strength of the association does not appear to be large, the lines show the direction of association for each measure. For the health efficacy measure, a positive association was observed with the number of clicks, and for all other measures the association appears to be flat or negative.

We analyzed each of the efficacy measures separately using a Poisson regression model with number of clicks as the outcome. Exponentiated coefficients from these models can be interpreted as rate ratios and are provided in Table 4, along with 95% confidence intervals and *P* values. Most efficacy measure scores at baseline were not significantly associated with the number of clicks. However, help-seeking behavior was highly significant (*P*<.001), with a rate ratio of 0.82 (0.73, 0.92), indicating that each 1-unit increase in help-seeking score at baseline is associated with an 18% decrease in the expected number of clicks throughout the study. Alternatively, one could say that someone with a help-seeking score of 4 at baseline has, on average, 0.82 times the number of clicks of someone with a help-seeking score of 5 at baseline. Higher help-seeking scores at baseline indicate those who are more likely to seek help for themselves or others; this relationship indicates that having a self-described higher amount of help-seeking behavior at baseline may actually make someone less likely to click frequently. This is a surprising finding.

**Table 4:** Poisson regression results.^a^

Measure	Rate ratio (95% CI)	*P* value
Health	1.02 (0.93, 1.12)	.74
Resilience	0.89 (0.78, 1.02)	.10
Negative coping	0.99 (0.91, 1.08)	.81
Positive coping	0.95 (0.86, 1.06)	.38
Self-efficacy	0.89 (0.79, 1.01)	.07
Self-esteem	0.90 (0.81, 1.01)	.07
Cultural identity	0.91 (0.8, 1.03)	.14
Help-seeking behavior	0.82 (0.73, 0.92)	<.001

^a^Each row in the table represents results from a Poisson regression model with the number of clicks as the outcome and the indicated BRAVE measure as the covariate of interest.

## Discussion

### Principal Findings

This paper offers an approach for passively measuring user engagement with a technology-based intervention to support mental health among AI/AN youth. The contributions of the paper are twofold; first, the methods contribute specific strategies for measuring engagement in technology-based health promotion interventions, an important contribution that offers a partial solution to address the limited agreement on how to measure and document engagement with technology-based solutions to promote health. Second, we offer data that help elucidate the breadth and impact of engagement for a nationwide mHealth intervention that successfully recruited participants from a hard-to-reach group, Native youth.

We offer specific strategies to document engagement with an SMS text messaging campaign, including documenting the total number of messages reviewed, time of day, day of the week, and immediacy of engagement (ie, how soon after messages are distributed are they read?). The findings of this paper illustrate important engagement outcomes that can be useful to establish realistic expectations for the level of intensity and population reach for a health-related SMS text messaging campaign. Few SMS text messaging interventions track engagement specifically and then conduct analyses that explore the impact of greater engagement on outcomes. We believe doing so is becoming easier to accomplish and can offer greater programmatic insight into SMS content, intensity, and series length that can become standard measures in future program implementation.

Factors that contribute to differences in user engagement should be explored in future studies. Contextual factors that may explain user engagement include social and environmental priorities within Native populations. Individual and community-level factors that likely influence engagement include phone ownership, network access, cost of devices, network infrastructure, and location [34]. One SMS intervention to support healthy lifestyle interventions among AI families reported different engagement levels based on urban and rural status, where urban participants liked and commented on posts more than rural participants [35] Phone number changes may also account for lower engagement. The Healthy Children, Strong Families 2 study reported that nearly one-third of AI participants changed their phone number during the intervention [35].

Although there was only modest engagement with the clickable SMS messages, it is of particular interest to note the findings that demonstrate a positive association between greater engagement with messages and self-reported help-seeking behavior, suggesting that engagement with 3 or more messages has a positive impact on overall help-seeking behavior for participants. This is among a very limited number of studies we are aware of that is able to elucidate a particular number of engagements that is needed to generate benefit, whether for in-person or virtual interventions [21]. The data showing that greater self-reported help-seeking behavior is associated with less engagement are challenging to interpret. It may be that people with a greater sense of efficacy seeking help at baseline did not consider the BRAVE messages personally relevant as they already had tools they employed to support their mental health and wellbeing.

As the use of SMS text messaging for health promotion becomes more commonplace, having standards for measuring engagement should become more accepted and is expected to help in the evaluation of these interventions. In addition, we believe that more dose-finding studies are needed to ascertain the optimal message delivery for mHealth text messages interventions like BRAVE [15,16,36]. BRAVE was designed to engage users with bidirectional messaging. Previous studies report positive outcomes from 1-way SMS text messaging interventions, including increased clinical appointments, childhood vaccinations, and malaria control [37,38]. SMS interventions are particularly useful for low- to middle-income countries and marginalized groups in the United States because users do not pay for incoming messages. Cost and user considerations should be carefully weighed when designing SMS interventions for these populations.

We assert that our methods have helped to set initial standards for what can be measured, while offering some caution for the limitations of these measures given current technological capabilities for SMS text messaging campaigns. Importantly, our work underscores the feasibility of exploring the impact of engagement on intended outcomes, allowing for more precise exploration of the dose-response relationship that may be realized through these low-touch initiatives with potential for reaching high numbers of users.

### Limitations

There are several limitations of this study. Our sample size was small for some of the engagement measures and some of the data were not easily captured. For example, some phone numbers from clicks were not linked to participants who had completed follow-up surveys, so for these participants, we could not explore a relationship between engagement and demographic or efficacy data. By contrast, for some participants, we had efficacy and demographic data, but no engagement data; thus, we could not determine if the lack of engagement (0 clicks) was truly due to no engagement or because we were not able to recover engagement data for those users. Finally, for participants who interacted with the content by going to the site of the material directly (eg, accessing a video on YouTube) rather than clicking on the link in the text message, we were not able to track content accessed in this way. Overall, the engagement data is likely an underestimation. This suggests having multiple approaches to tracking data that go beyond the passive methods we described here could be an improvement in study design. There are also systems that integrate the backend databases and engagement logs, so that all engagement is tracked through the same system that delivered messages, such as Twilio (Twilio Inc) [39] or Messenger (Meta Platforms Inc) [40], two commercial platforms for scaled text message delivery campaigns.

### Conclusions

The BRAVE intervention was designed and promoted by the We R Native team, whose extensive credibility and reach with AI/AN youth was potentially a factor in having users read messages quickly (eg, half of users reading messages within the first minute of distribution) and in having users access content (primarily YouTube videos developed by the team). Despite this credibility, there was still a very modest level of engagement overall, with users only clicking a median of 2 message links from the campaign. This suggests that it may be challenging, even for a well-known and well-respected organization that has strong ties to the communities it serves, to use text messages as a stand-alone intervention strategy.

Although there have been positive outcomes from stand-alone SMS text messaging campaigns, such as those targeting smoking cessation and healthy pregnancy [41], the majority of positive outcomes from SMS text messaging interventions for health promotion have been linked to specific clinical or organizational interventions that facilitate amplification of clinic- or community-based initiatives with supplemental texting to enhance or extend that which occurs in face-to-face settings [13,42-44]. A useful next step would be to consider linking the BRAVE intervention to school-, clinic-, or community-based mental health programs for AI/AN youth as a way to enhance the intervention. Doing so could help build connections to local health services and destigmatize help-seeking, an important goal of the BRAVE SMS series.

## Abbreviations

AI/AN: American Indian and Alaska Native
NPAIHB: Northwest Portland Area Indian Health Board
SAMHSA: Substance Abuse and Mental Health Service Administration
TAM: Technology & Adolescent Mental Wellness
